# Dynamic intravascular ultrasound changes mimicking membrane disruption in a covered stent during management of coronary perforation

**DOI:** 10.1093/ehjcr/ytag607

**Published:** 2026-08-14

**Authors:** Yutaka Matsuhiro, Daisuke Nakamura, Keita Okayama, Yasushi Sakata

**Affiliations:** Department of Cardiovascular Medicine, The University of Osaka Graduate School of Medicine, 2-2 Yamadaoka, Osaka 565-0871, Japan; Department of Cardiovascular Medicine, The University of Osaka Graduate School of Medicine, 2-2 Yamadaoka, Osaka 565-0871, Japan; Department of Cardiovascular Medicine, The University of Osaka Graduate School of Medicine, 2-2 Yamadaoka, Osaka 565-0871, Japan; Department of Cardiovascular Medicine, The University of Osaka Graduate School of Medicine, 2-2 Yamadaoka, Osaka 565-0871, Japan

**Keywords:** PK Papyrus, Intravascular ultrasound, Coronary perforation

## Summary

A blowout-type coronary perforation occurred following rotational atherectomy at the left circumflex artery (LCX) ostium. Haemostasis was attempted with sequential deployment of PK Papyrus covered stents at the LCX ostium and from the left main trunk to the left anterior descending artery (LAD); however, contrast extravasation persisted. Serial intravascular ultrasound (IVUS) demonstrated progressive attenuation of the characteristic high-intensity stent signals, raising concern for covered membrane disruption. Additional covered stenting with a Graftmaster and PK Papyrus nevertheless failed to achieve complete sealing of the perforation.

## Case description

**Figure 1:**
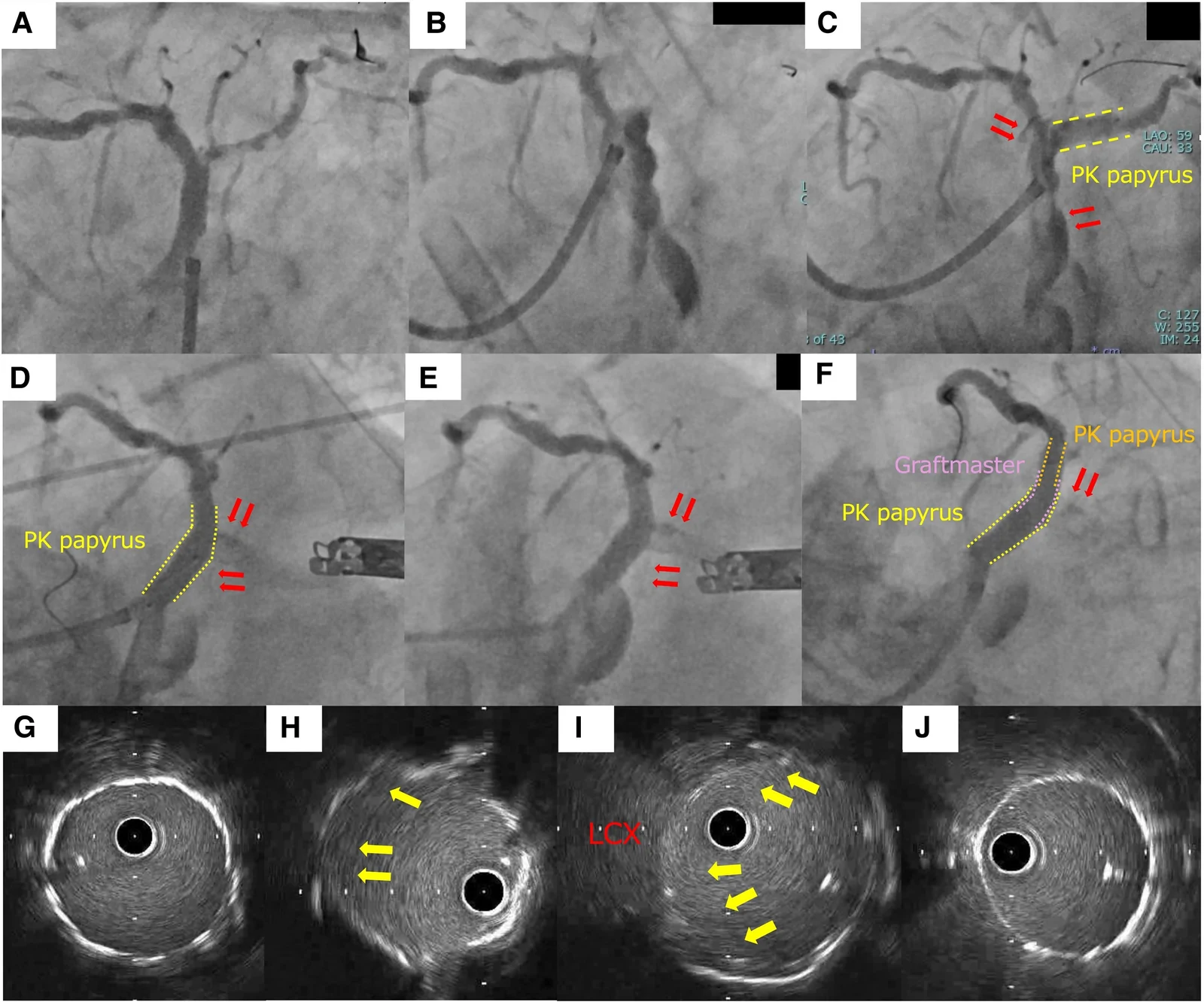
Serial angiographic and intravascular ultrasound findings. (*A*) Initial angiography. (*B*) Coronary perforation occurred at the left circumflex artery ostium. (*C*) Deployment of a PK Papyrus (3.5 × 20 mm) at the left circumflex artery ostium. (*D*) Additional deployment of a PK Papyrus (3.5 × 20 mm) from the left main trunk to the left anterior descending artery; however, contrast extravasation persisted. (*E*) Persistent contrast extravasation despite repeated post-dilatation using a 5 mm non-compliant balloon and a 1-h waiting period. (*F*) Additional covered stenting with a Graftmaster (3.5 × 16 mm) and a PK Papyrus (2.5 × 20 mm) failed to achieve complete sealing of the perforation. (*G*) Intravascular ultrasound image immediately after PK Papyrus deployment. Intravascular ultrasound imaging was performed using the AltaView system (Terumo, Tokyo, Japan) at 60 MHz with a pullback speed of 9 mm/s. The gain settings were G 15, C 3, Gm 7, S 1, and T 3. (*H*) Intravascular ultrasound image after post-dilatation using a 5 mm non-compliant balloon. (*I*) Intravascular ultrasound image after repeated post-dilatation and a 1-h waiting period. (*J*) Intravascular ultrasound image after Graftmaster deployment. Red arrows indicate contrast extravasation. The yellow arrow indicates an area of reduced conspicuity of the Papyrus membrane.

An 83-year-old woman with ischaemic cardiomyopathy and severe calcified lesions in the LAD and LCX underwent coronary artery bypass grafting; however, incomplete revascularization necessitated staged percutaneous coronary intervention (PCI). After successful stenting of the LAD, PCI to the LCX was attempted. Due to severe calcification, rotational atherectomy was performed using a 1.5-mm burr. During the procedure, a blowout-type coronary perforation occurred, resulting in cardiac arrest (*Figures 1A* and *B*). Veno-arterial extracorporeal membrane oxygenation and pericardial drainage were immediately initiated. Haemostasis was attempted with sequential deployment of PK Papyrus covered stents at the LCX ostium and from the left main trunk to the LAD; however, contrast extravasation persisted (*Figure 1C* and *D*). Despite repeated post-dilatation, leakage persisted (*Figure 1E*). The activated clotting time was maintained at 150–170 s during this period. Serial IVUS demonstrated progressive attenuation of the characteristic high-intensity stent signals on repeat imaging 1 h later, raising concern for covered membrane disruption (*Figures 1G–I*, see Supplementary material online, *Videos S1 and S2*). After additional deployment of a Graftmaster, the characteristic high-intensity membrane structure became clearly visible on IVUS (*Figure 1J* and Supplementary material online, *Video S3*). Subsequent deployment of an additional PK Papyrus in the distal segment failed to achieve complete sealing of the perforation (*Figure 1F*). One possible explanation is that the PK Papyrus covered stent contains numerous microscopic pores within its polyurethane membrane, allowing progressive deposition of blood components over time. This may have altered the IVUS appearance by reducing the contrast between the covered stent and the surrounding blood. However, this hypothesis remains speculative because direct assessment of the membrane was not possible in the present case. Fortunately, haemostasis was eventually achieved conservatively after administration of fresh frozen plasma to reverse the markedly prolonged activated partial thromboplastin time, and the patient was successfully weaned from extracorporeal support.

This case demonstrated dynamic changes in the IVUS image of PK Papyrus mimicking membrane disruption. Given the increasing use of this device in contemporary practice, sharing these distinctive imaging characteristics may be of substantial clinical relevance.

## Supplementary Material

ytag607_Supplementary_Data

## Data Availability

The data underlying this article will be shared on reasonable request to the corresponding author.

## Notes

**Consent:** The authors confirm that consent for the submission and publication of this work has been obtained, in accordance with the COPE guidelines.

