# Supplementary material for: Dynamic intravascular ultrasound changes mimicking membrane disruption in a covered stent during management of coronary perforation
Source: Eur Heart J Case Rep. 2026 Aug 14;10(9):ytag607. doi: 10.1093/ehjcr/ytag607 (PMC13524320; doi:10.1093/ehjcr/ytag607)
Supplement: Supplementary file 1 [file ytag607_supplementary_data.zip › Supplemental video legends.docx]

**Video 1**

**Title:** IVUS immediately after PK Papyrus deployment

**Video 2**

**Title:** IVUS after 5 mm post-dilation and a 1-hour waiting period

**Video 3**

**Title:** IVUS after Graftmaster deployment
